# Long-Term Survival Heterogeneity Across PD-L1-Defined Subgroups in Pembrolizumab-Treated Non-Small-Cell Lung Cancer: A Model-Based Analysis

**DOI:** 10.3390/pharmaceutics18091177

**Published:** 2026-09-17

**Authors:** Heungjo Kim, Sungjae Lee, Hongjae Lee, Ji Woo Lim, Tahir Sher, Chanwoo Kim, Junjeong Choi, Sun Min Lim, Min Jung Chang

**Affiliations:** 1 Department of Pharmacy and Yonsei Institute of Pharmaceutical Sciences, College of Pharmacy, Yonsei University, Incheon 21983, Republic of Korea; heungjo@yonsei.ac.kr (H.K.); chao1984@naver.com (S.L.); manu5315@yonsei.ac.kr (H.L.); lmjw@yonsei.ac.kr (J.W.L.); junjeong@yonsei.ac.kr (J.C.); 2 Department of Pharmaceutical Medicine and Regulatory Science, Yonsei University, Incheon 21983, Republic of Korea; 3 Department of Integrative Biotechnology, College of Pharmacy, Yonsei University, Incheon 21983, Republic of Korea; 4 Department of Artificial Intelligence, Korea University, Seoul 02841, Republic of Korea; tahir2011.iiui@gmail.com (T.S.); chanwcom@korea.ac.kr (C.K.); 5 Graduate Program of Industrial Pharmaceutical Science, Yonsei University, Incheon 21983, Republic of Korea; 6 Division of Medical Oncology, Department of Internal Medicine, Yonsei University College of Medicine, Seoul 03722, Republic of Korea; limlove2008@yuhs.ac; 7 College of Pharmacy, Yonsei-SL Bigen Research Institute, Yonsei University, Incheon 21983, Republic of Korea

**Keywords:** non-small-cell lung cancer, pembrolizumab, parametric modeling, survival prediction

## Abstract

**Background/Objectives**: Immune checkpoint inhibitors have substantially improved survival outcomes in non-small-cell lung cancer (NSCLC). However, survival heterogeneity across PD-L1-defined and other clinically relevant patient subgroups remains incompletely characterized, limiting subgroup-specific interpretation of pembrolizumab outcomes within a precision oncology framework. In this study, we quantified survival heterogeneity and estimated long-term outcomes of pembrolizumab using reconstructed clinical trial data. **Methods**: A systematic review identified 36 trials in advanced NSCLC. Individual time-to-event and censoring records were reconstructed from published Kaplan–Meier curves, while patient-level clinical and demographic covariates were stochastically assigned according to trial-level summary distributions. Parametric time-to-event models were developed for overall survival (OS) and progression-free survival (PFS) across programmed death-ligand 1 (PD-L1) tumor proportion score subgroups. Model performance was evaluated using bootstrap analyses and external validation, and model-based simulations were conducted to project survival for up to 10 years. **Results**: A total of 7165 reconstructed time-to-event records for OS and 6730 for PFS were included in the analyses. Model-predicted survival outcomes varied across clinically relevant characteristics, with higher PD-L1 expression, treatment-naïve status, non-squamous histology, and combination therapy generally associated with more favorable outcomes. Long-term projections demonstrated substantial heterogeneity across covariate-defined subgroups, with projected 10-year OS probabilities ranging from 7.2% to 18.0% among treatment-naïve subgroups. **Conclusions**: These findings provide a quantitative characterization of subgroup-specific survival heterogeneity and long-term outcomes in pembrolizumab-treated NSCLC, supporting a more refined interpretation of treatment outcomes in the context of precision oncology.

## 1. Introduction

Lung cancer remains the leading cause of cancer-related mortality worldwide, accounting for approximately 1.8 million deaths annually [1,2]. Non-small-cell lung cancer (NSCLC) represents approximately 85% of all lung cancer cases, with most patients diagnosed at an advanced stage (IIIB–IV), where curative treatment options are limited [3,4]. Historically, platinum-based chemotherapy provided only modest clinical benefit, with median overall survival (OS) measured in months and 5-year survival rates rarely exceeding 5–10% [5,6].

The introduction of immune checkpoint inhibitors (ICIs), particularly pembrolizumab targeting the PD-1/PD-L1 pathway, has substantially improved outcomes in NSCLC. By restoring T-cell–mediated antitumor activity through inhibition of PD-1 signaling, pembrolizumab has demonstrated consistent survival benefits across multiple clinical settings [7]. Following its initial approval for previously treated PD-L1–positive disease, pembrolizumab rapidly expanded into the first-line setting [8,9]. Landmark trials, including KEYNOTE-024, KEYNOTE-189, and KEYNOTE-407, established its efficacy both as monotherapy in patients with high PD-L1 expression and in combination with chemotherapy across histologic subtypes [10,11,12,13]. These studies have positioned pembrolizumab as a cornerstone of modern NSCLC therapy.

Despite these advances, the extent to which long-term pembrolizumab outcomes vary across clinically relevant patient subgroups remains incompletely characterized. Such variability may be associated with clinical and molecular characteristics, including PD-L1 TPS, histology, prior treatment status, and EGFR mutation, as well as broader mechanisms of tumor evolution and therapeutic resistance [14,15]. Extended follow-up from trials such as KEYNOTE-024 and KEYNOTE-042 has demonstrated sustained benefit, with 5-year OS rates of approximately 25–32% in patients with high PD-L1 expression [10,16,17]. Recent real-world studies have reported comparable outcomes, with 5-year OS rates of 24–25% in clinical practice [18,19]. Earlier-phase studies, including KEYNOTE-001, suggest that a subset of patients may achieve durable survival well beyond 5 years; however, a comprehensive characterization of long-term outcomes is still lacking [8,20]. This gap is particularly relevant in the context of ICIs, which can produce long-term survival plateaus in a subset of patients [21,22,23].

Although multiple pembrolizumab trials have reported OS and progression-free survival (PFS), the available evidence remains fragmented across treatment settings, PD-L1 subgroups, histologic populations, and follow-up durations. In addition, most publications provide only aggregate survival summaries or Kaplan–Meier (KM) curves, which limits integrated evaluation of long-term survival patterns across clinically defined populations. As a result, the extent of survival heterogeneity and the expected range of long-term outcomes remain difficult to quantify from individual trial reports alone.

Conventional nonparametric survival analyses, such as KM estimation, are well suited for summarizing observed outcomes but offer limited insight into survival heterogeneity and long-term extrapolation [24]. In contrast, model-based time-to-event (TTE) approaches provide a quantitative approach for characterizing survival distributions, comparing clinically defined subgroups, and projecting outcomes beyond the observed trial period [25,26,27]. Such approaches are particularly relevant in immuno-oncology, where long-term survival tails and nonconstant hazard patterns may not be adequately represented by short-term summary measures alone [28].

In this study, we addressed these limitations by reconstructing individual patient-level data (IPD) from published pembrolizumab trials and integrating them into a pooled analytical framework. Using parametric TTE modeling, we quantified survival heterogeneity across PD-L1 tumor proportion score subgroups and other key covariates and projected long-term OS probabilities at 5, 7, and 10 years under clinically relevant scenarios. These findings provide a quantitative characterization of subgroup-specific survival heterogeneity and long-term outcomes in pembrolizumab-treated NSCLC, supporting a more refined interpretation of treatment outcomes in the context of precision oncology. From a pharmacometric and model-informed drug development perspective, this framework also illustrates how reconstructed clinical trial data can be integrated to characterize synthesized data-driven variability and extend survival assessment beyond the observed follow-up period.

## 2. Methods

### 2.1. Literature Search and Study Selection

A systematic literature search was performed using PubMed, Embase, and the Cochrane Library, from database inception to 11 February 2025, to identify clinical trials evaluating pembrolizumab in patients with NSCLC. Eligible studies were clinical trials of pembrolizumab, either as monotherapy or in combination with other systemic anticancer therapy, that reported KM curves for OS and/or PFS, irrespective of treatment line or control arm. Duplicate records identified across databases were removed before title and abstract screening, followed by full-text assessment of potentially eligible studies.

Following full-text review, studies were excluded if they involved overlapping patient populations, represented pooled or secondary analyses, or lacked extractable KM data, or otherwise did not meet the predefined eligibility criteria. When multiple publications reported the same trial population at different follow-up durations, only the publication with the longest available follow-up for the relevant treatment arm was retained to avoid duplicate inclusion of the same patients. Detailed reasons for exclusion at the full-text stage and the corresponding numbers of excluded studies are presented in the PRISMA flow diagram (Figure S1), and the PICOS-SD framework and eligibility criteria are provided in Table S1.

This review was conducted in accordance with the PRISMA 2020 guidelines [29]. Risk of bias was assessed using the Cochrane Risk of Bias 2.0 tool [30] for randomized controlled trials and the Methodological Index for Non-Randomized Studies [31] for single-arm or non-randomized trials.

### 2.2. Data Extraction

**Figure 1:**
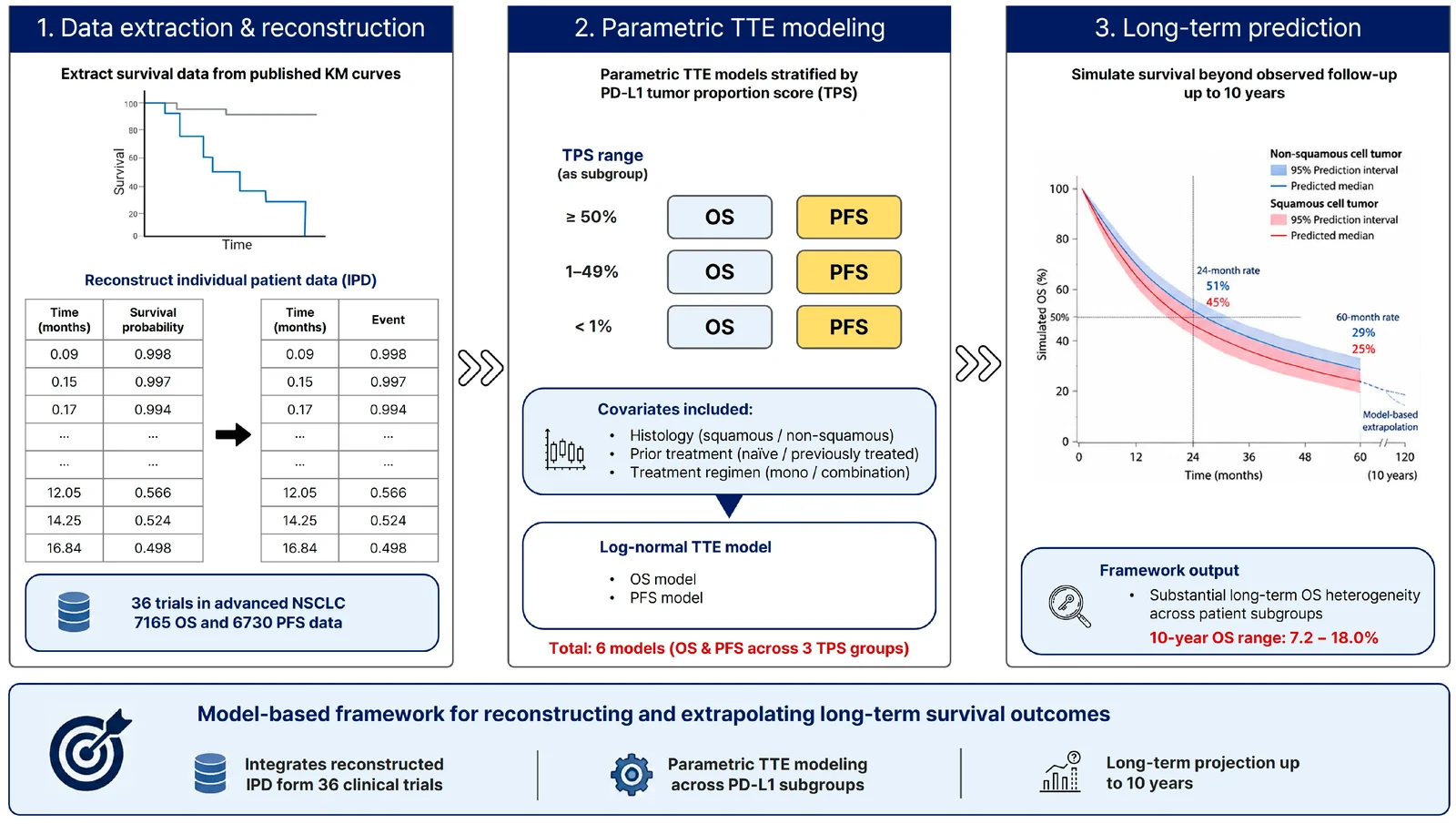
Overall workflow of reconstructed individual patient-level data-based survival modeling. Schematic overview of the analytical framework used for reconstructed individual patient-level data-based survival modeling. Published Kaplan–Meier curves for overall survival and progression-free survival from eligible pembrolizumab clinical trials were digitized and reconstructed into individual patient-level data. Parametric time-to-event models were subsequently developed across PD-L1 tumor proportion score subgroups, incorporating relevant clinical covariates. The final models were evaluated through external validation and applied for long-term survival simulations and subgroup-specific survival projections.

For each eligible trial, KM curves for OS and PFS were digitized and reconstructed into IPD using the IPDfromKM web tool [32]. Published KM curves were digitized to extract time–survival coordinates, and reported numbers at risk, when available, were incorporated into the reconstruction procedure. Event and censoring times were reconstructed within intervals defined by the reported numbers at risk, following the algorithm implemented in IPDfromKM. The accuracy of each reconstructed IPD was verified using the diagnostic statistics provided by the IPDfromKM tool, including root mean square error, mean absolute error, and the Kolmogorov–Smirnov test. The overall analytical workflow is summarized in Figure 1. The reconstructed IPD comprised TTE outcomes (event or censoring) and was enriched with study-level covariates derived from the demographic characteristics reported in the original publications. Covariates included age (continuous), sex, race, Eastern Cooperative Oncology Group (ECOG) performance status, smoking history, histology (squamous vs. non-squamous), presence of metastasis (including brain metastasis), PD-L1 TPS (< 1%, 1–49%, or ≥ 50%), prior systemic therapy status (treatment-naïve vs. previously treated), treatment regimen (monotherapy vs. combination therapy), with combination therapy defined as any pembrolizumab-containing regimen administered together with an additional systemic anticancer agent, including platinum-based chemotherapy, ipilimumab, ramucirumab, or lenvatinib, and epidermal growth factor receptor (EGFR) mutation status. When only summary statistics (e.g., proportions, medians, ranges) were available, representative values were assigned to approximate the distribution of baseline characteristics within each trial population. Because this assignment process involves stochastic imputation, we generated 10 independent datasets and evaluated pairwise differences in covariate distributions using Cliff’s delta, a nonparametric effect size measure [33].

### 2.3. TTE Model Development

TTE models were developed to characterize the survival profiles of patients with NSCLC treated with pembrolizumab. Separate models were constructed for OS and PFS; all analyses were performed using MonolixSuite (version 2024R1; Lixoft, Antony, France). Parameter estimation was conducted using the stochastic approximation expectation–maximization algorithm. To address heterogeneity by PD-L1 TPS, three stratified models (<1%, 1–49%, ≥50%) were developed, alongside a unified model incorporating TPS as a categorical covariate to evaluate the feasibility of pooled analysis. Interindividual variability (IIV) was modeled using a log-normal distribution: *θ_i_* = *θ_Pop_* × EXP(*η_i_*), where *θ_i_* is the individual parameter value, *θ_Pop_* is the population estimate, and *η_i_* is a normally distributed random effect with mean zero and variance *ω_η_*_2_ [34].

To identify the most appropriate structural model, five parametric survival distributions were evaluated: Weibull, Gompertz, generalized gamma, log-logistic, and log-normal [35,36,37]. Candidate distributions were compared using the Akaike information criterion (AIC) and Bayesian information criterion (BIC), with lower values indicating a better balance between model fit and complexity. Differences in AIC were interpreted using conventional thresholds, with ΔAIC < 2 indicating comparable support between models and ΔAIC >10 indicating substantially weaker support for the model with the higher AIC. Model selection was further supported by visual goodness-of-fit diagnostics and model stability. Each distribution was defined by its survival function $S\left(t\right)$, representing the probability of surviving beyond time t. The corresponding survival functions are provided in Table S2.

### 2.4. Covariate Model Development

A covariate model was developed to assess the effect of clinical and demographic factors on survival outcomes. Stepwise covariate modeling, consisting of forward inclusion and backward elimination, was used to identify significant predictors. Candidate covariates included age (continuous) and categorical variables such as sex, race, ECOG performance status, smoking history, histology, metastatic status (including brain metastasis), PD-L1 TPS, prior systemic therapy status, treatment regimen, and EGFR mutation status.

Continuous covariates were tested under power, exponential, and proportional models, with variables centered at their median values, while categorical covariates were implemented as indicator variables. Model selection was guided by reductions in objective function value (OFV) and AIC, model stability, and VPCs. Statistical significance between nested models was assessed using the likelihood ratio test, with decreases in OFV of ≥3.84 (forward inclusion) and ≥6.63 (backward elimination) considered significant, corresponding to *p* < 0.05 and *p* < 0.01, respectively.

(1) $$\mu =\mu_{pop}\cdot exp\left(\sum_{k=1}^{n}\beta_{\mu ,k}\cdot {COV}_{k}+\eta_{\mu }\right)$$

(2) $$\sigma =\sigma_{pop}\cdot exp\left(\eta_{\sigma }\right)$$

where *μ* and *σ* denote the location and dispersion parameters of the log-normal distribution, $\beta_{\mu ,k}$ represents the covariate effect, and η is a random effect with mean zero and variance $\omega^{2}$.

### 2.5. Model Evaluation

The performance of the final models was assessed using multiple diagnostic approaches. VPCs were conducted by comparing observed estimates for OS and PFS with model-simulated survival distributions, including median trajectories and 5th/95th prediction percentiles. The robustness and precision of parameter estimates were examined through a nonparametric bootstrap procedure with 5000 replicates, from which CIs were derived.

To assess cross-trial variability within clinically comparable populations, additional subgroup-level heterogeneity analyses were performed using cohorts with directly reported survival outcomes. Analyses were restricted to cohorts sharing similar PD-L1 status, treatment line, and pembrolizumab treatment type. Pooled median OS estimates were derived from the reported median survival and corresponding 95% confidence intervals, and between-study heterogeneity was assessed using Cochran’s Q test.

### 2.6. External Validation

To evaluate the generalizability of the developed TTE models, external validation was conducted using pembrolizumab clinical trials that were not included in the original SR used for IPD reconstruction. A targeted literature search was performed to identify relevant trials published after 12 February 2025, using the same search strategy.

For each external study, the previously developed parametric survival models were applied in a forward-prediction method by aligning model covariates to the reported baseline characteristics and subgroup proportions for the target trial. Using these covariate distributions, individual-level event times were generated based on the fitted hazard functions to obtain predicted survival profiles at the population level.

Model-derived survival curves were then compared with the corresponding published KM curves from the external trials. For quantitative evaluation, survival probabilities at 12, 24, and 36 months were extracted from both the model-derived predictions and the external KM curves.

### 2.7. Simulation

**Figure 2:**
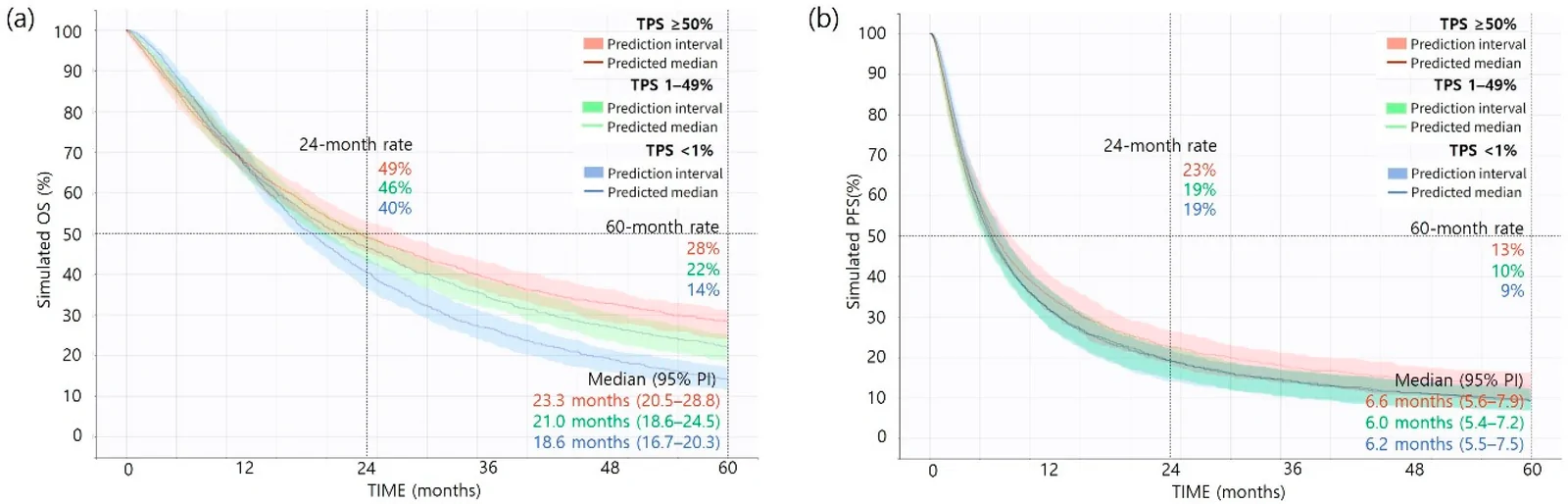
Simulated overall survival and progression-free survival curves according to tumor proportion score models. Simulated OS (**a**) and PFS (**b**) based on final models constructed separately by tumor PD-L1 expression (TPS ≥ 50%, 1–49%, <1%). Solid lines indicate median survival; shaded areas represent 95% prediction intervals. Red, blue, and green denote TPS ≥ 50%, 1–49%, and <1% groups, respectively. Each panel includes simulated 24- and 60-month survival rates and median survival times with 95% PIs. OS, overall survival; PFS, progression-free survival; TPS, tumor proportion score; PI, prediction interval.

To explore long-term survival outcomes beyond the available follow-up, model-based simulations were conducted using the final parametric OS and PFS models. Survival outcomes were characterized separately for each PD-L1 TPS category (<1%, 1–49%, and ≥50%). Subgroup simulations were based only on covariates retained in the corresponding final TPS-specific model, which included prior systemic therapy status, histological subtype, or treatment regimen depending on the model. Long-term OS probabilities at 5, 7, and 10 years were estimated under clinically relevant covariate scenarios. For the comparison of survival across PD-L1 TPS categories (Figure 2), the covariates retained in each final model were standardized, with continuous covariates fixed at their median values and categorical covariates assigned a 1:1 distribution, so that survival could be compared under otherwise comparable covariate conditions.

## 3. Results

### 3.1. Characteristics of Eligible Studies

From 2770 records initially screened, 36 clinical trials met the inclusion criteria for TTE modeling. The study selection process is shown in the PRISMA flow diagram (Figure S1). Detailed study characteristics, together with bibliographic references for each trial, are provided in Table S3. Results of the risk of bias assessment are presented in Figure S2 and Table S4.

### 3.2. Characteristics of Reconstructed IPD for TTE Modeling

Baseline characteristics of the reconstructed IPD are summarized in Table 1. A total of 7165 and 6730 patient records were generated for OS and PFS modeling, respectively. Cliff’s delta analysis across the 10 independently imputed datasets indicated small effect sizes (|*δ*| = 0.15), suggesting that the stochastic covariate assignment process did not substantially alter the overall covariate distributions. The study population was broadly representative of advanced NSCLC, with a mean age of 64 years; most patients were male, non-Asian, and had a history of smoking. Non-squamous histology predominated, and the majority of patients were treatment-naïve at enrollment. In the OS cohort, PD-L1 expression was broadly distributed, with the highest frequency in the TPS ≥ 50% group.

Among eight treatment-naïve cohorts with PD-L1 TPS ≥ 50% receiving pembrolizumab monotherapy, median OS ranged from 19.9 to 29.6 months, with a pooled median OS of 22.6 months (95% CI, 20.6–24.7). Cochran’s Q was 4.22 (*p* = 0.754), indicating no statistically significant evidence of between-study heterogeneity. Similarly, among four cohorts with PD-L1 TPS 1–49% receiving pembrolizumab monotherapy, median OS ranged from 12.6 to 18.6 months, with a pooled median OS of 15.3 months (95% CI, 12.4–18.9; Q = 4.91, *p* = 0.179).

### 3.3. Parametric TTE Modeling Outcomes

Separate TTE models for OS and PFS were developed across the three TPS categories (<1%, 1–49%, and ≥50%). Among the candidate parametric distributions, the log-normal model consistently provided the best fit, as indicated by reductions in the AIC and objective OFV. Fixed- and random-effect parameter estimates for the TPS ≥ 50% and 1–49% models are presented in Table 2, and the results for the TPS < 1% model are provided in Table S5. Covariate and random effects were incorporated through exponential relationships, as specified in Equations (1) and (2).

In the OS models, the TPS ≥ 50% group yielded the highest μ estimate (2.58; 95% confidence interval [CI], 2.45–2.72). Prior systemic therapy status was consistently retained as a covariate across all models, with treatment-naïve patients associated with higher μ values. Non-squamous histology was additionally included in the TPS ≥ 50% and <1% models and combination therapy was retained in the TPS 1–49% model (*β* = 0.13; 95% CI, 0.08–0.18) (Table 2). No other candidate covariates met the predefined criteria for retention in the final OS models.

In the PFS models, treatment-naïve status was associated with higher μ values across all models. In the TPS ≥ 50% model, non-squamous histology was retained (*β* = 0.1; 95% CI 0.03–0.17) (Table 2). The visual predictive check (VPC) demonstrated that the observed survival curves for OS and PFS were well-captured within the simulated prediction intervals (Figure S3). No other candidate covariates met the predefined criteria for retention in the final PFS models.

### 3.4. Outcome of External Validation

Two external clinical trials (LEAP-006 and LEAP-008) were identified for validation (Table S6). In LEAP-006, the placebo-control arm consisting of pembrolizumab plus chemotherapy (*n* = 373) was used for external validation. In LEAP-008, the control arm receiving lenvatinib plus pembrolizumab (*n* = 185) was included for validation analyses [38,39].

In LEAP-006, predicted survival probabilities at 12, 24, and 36 months closely matched the observed estimates, with absolute differences of approximately 2% or less. In LEAP-008, the model modestly overestimated 12-month survival (51.3% predicted vs. 46.5% observed), while predictions at 24 and 36 months remained closely aligned with the reported outcomes (Table 3).

### 3.5. Simulation Outcomes According to TPS-Specific Models

Simulations based on the final TTE models were conducted separately for each PD-L1 TPS category (Figure 2), with other modeled covariates standardized to representative values. In the OS simulations, the projected median survival was 23.3 months (95% PI, 20.5–28.8) in the TPS ≥ 50% group, 21.0 months (95% PI, 18.6–24.5) in the TPS 1–49% group, and 18.6 months (95% PI, 16.7–20.3) in the TPS < 1% group.

In the PFS simulations, median survival was 6.6 months (95% PI, 5.6–7.9) in the TPS ≥ 50% group, 6.0 months (95% PI, 5.4–7.2) in the TPS 1–49% group, and 6.2 months (95% PI, 5.5–7.5) in the TPS < 1% group. Although the TPS ≥ 50% group had the longest median PFS, the 95% PIs overlapped across all groups.

### 3.6. Simulation Outcomes According to Covariates

**Figure 3:**
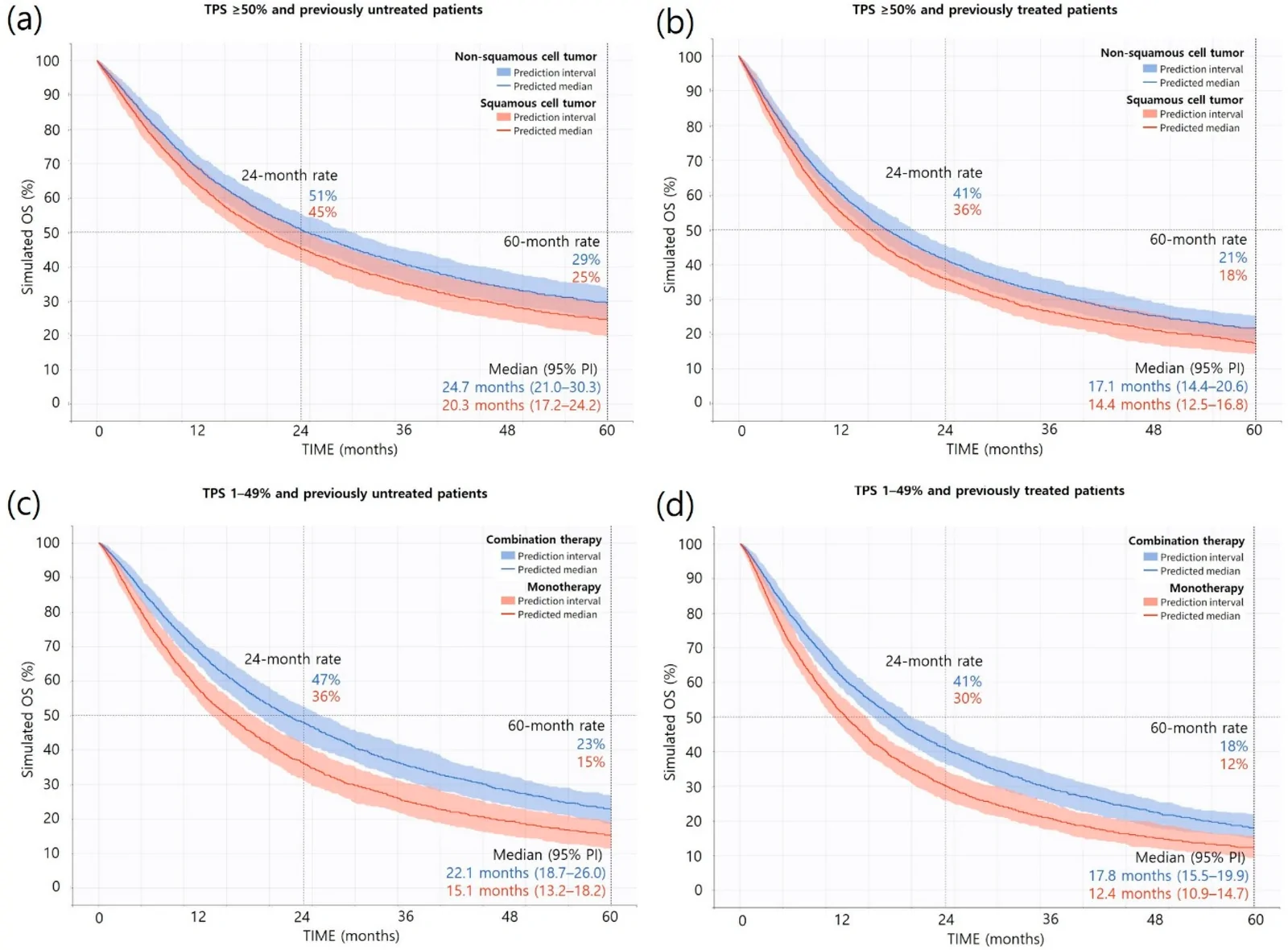
Simulated overall survival curves stratified by histology and treatment regimen based on covariate-specific final models. (**a**,**b**) TPS ≥ 50% model stratified by histology for previously untreated (**a**) and treated (**b**) patients. (**c**,**d**) TPS 1–49% model stratified by treatment regimen for untreated (**c**) and treated (**d**) patients. Blue and red shaded areas represent 95% PIs for non-squamous vs. squamous (**a**,**b**) and combination vs. monotherapy (**c**,**d**), respectively. Solid lines indicate median OS. The 24- and 60-month survival rates and median OS (95% PI) are annotated. OS, overall survival; TPS, tumor proportion score; PI, prediction interval.

Covariate-based simulations were conducted to evaluate how specific clinical characteristics affected survival predictions. In the TPS ≥ 50% OS model (Figure 3a,b), treatment-naïve patients with non-squamous histology had a longer median OS (24.7 months; 95% PI 21.0–30.3) than those with squamous histology (20.3 months; 95% PI, 17.2–24.2). In the TPS 1–49% OS model (Figure 3c,d), combination therapy yielded a higher median OS than monotherapy across treatment-naïve and previously treated populations. For instance, in previously treated patients, combination therapy resulted in a higher median OS (17.8 months; 95% PI, 15.5–19.9) than monotherapy (12.4 months; 95% PI, 10.9–14.7). In the TPS < 1% OS model, histology remained a key determinant of outcome, with non-squamous tumors showing a consistently longer OS than squamous tumors, irrespective of prior treatment status. Detailed results are provided in Figure S4.

Covariate effects were also evident in the PFS models. In the TPS ≥ 50% model (Figure S5a,b), treatment-naïve patients with non-squamous histology achieved a longer median PFS (8.5 months; 95% PI, 7.4–10.2) than those with squamous histology (7.0 months; 95% PI, 6.0–8.5). In the TPS 1–49% model (Figure S5c), treatment-naïve patients had a longer median PFS (8.0 months; 95% PI, 6.9–9.6) than previously treated patients (4.7 months; 95% PI, 4.1–5.3). For the TPS < 1% model, PFS simulations stratified by prior treatment status are presented in Figure S6.

### 3.7. Long-Term OS Simulations

Long-term OS probabilities were projected using the final models for TPS ≥ 50% and TPS 1–49% (Table 4). Among treatment-naïve patients, the most favorable outcome was observed in the TPS ≥ 50% non-squamous subgroup, with 5-, 7-, and 10-year OS probabilities of 29% (95% PI, 24–33), 23% (95% PI, 19–28), and 18% (95% PI, 14–21). In contrast, the poorest outcome was observed in patients in the TPS 1–49% subgroup receiving monotherapy, with a 10-year OS probability of 7.2% (95% PI, 4.9–8.7). The results for previously treated populations are presented in Table S7. Consistent with the results in treatment-naïve patients, the TPS ≥ 50% non-squamous subgroup showed the most favorable prognosis, with a 10-year OS probability of 12% (95% PI, 9–16).

## 4. Discussion

This study provides a quantitative framework for integrating clinical trial data and estimating long-term survival beyond the observed follow-up period. By pooling reconstructed IPD from 36 clinical trials comprising more than 7000 patient records, we developed parametric TTE models that reproduced key survival trends reported in pivotal trials and enabled covariate-specific projections across PD-L1 TPS categories, histologic subtypes, treatment regimens, and prior treatment settings. This approach extends the available clinical trial evidence by providing a quantitative characterization of long-term survival heterogeneity in pembrolizumab-treated NSCLC.

We developed six separate parametric models for OS and PFS across the PD-L1 TPS < 1%, 1–49%, and ≥50% subgroups because the stratified models provided a better fit than a unified model incorporating TPS as a categorical covariate. Nonparametric bootstrap analyses indicated stable parameter estimation, and simulated survival profiles were consistent with observed trial data. Model-based simulations also yielded results comparable to those reported in pivotal clinical trials. For example, in the TPS ≥ 50% subgroup, the estimated median OS was 23.3 months (95% CI, 20.5–28.8), closely aligning with the outcomes reported in KEYNOTE-598 (21.9 months; 95% CI, 18.0–NR) and KEYNOTE-042 (20.0 months; 95% CI, 15.4–24.9) [16,40]. Moreover, the OS predictions reflected the established relationship between PD-L1 expression and clinical outcomes, with the TPS ≥50% subgroup showing the most favorable long-term survival profile.

Although OS estimates varied across TPS categories, PFS outcomes were largely overlapping. Median PFS was similar in the TPS 1–49% and <1% groups (6.0 vs. 6.2 months), suggesting that the association between PD-L1 expression and PFS may be less pronounced than that observed for OS. In pivotal trials, higher TPS has generally been associated with improved OS, whereas differences in PFS have been less consistent [16,41]. For example, the KEYNOTE-789 showed a difference in OS between high- and lower-TPS groups (18.6 vs. 15.7 months) but essentially overlapping PFS outcomes (5.6 months in both groups) [41]. PFS may also be more susceptible than OS to censoring, assessment schedules, and variability in radiographic evaluation. Reflecting these considerations, the U.S. Food and Drug Administration has recently emphasized OS as a preferred primary endpoint in oncology trials because of its objectivity, clinical relevance, and ability to capture both therapeutic benefit and potential harm [42]. Accordingly, OS was selected as the primary endpoint for long-term survival projections in this study.

Simulation analyses demonstrated clinically interpretable differences across covariate-defined subgroups. Among patients with TPS ≥ 50%, projected OS and PFS did not differ substantially between squamous and non-squamous histology, irrespective of prior treatment status. This pattern is consistent with current National Comprehensive Cancer Network (NCCN) guidelines (version 7.2025), which recommend pembrolizumab as first-line therapy for advanced NSCLC with TPS ≥ 50% across histologic subtypes [43]. In contrast, among patients with TPS 1–49%, combination therapy consistently yielded a longer projected median OS than monotherapy, regardless of prior treatment history. Notably, treatment regimen was retained as a significant covariate in the TPS 1–49% OS model but not in the TPS < 1% model. In the latter subgroup, where the contribution of pembrolizumab monotherapy is generally less pronounced, a distinct regimen-associated effect may have been more difficult to quantify within the pooled parametric framework. These findings were generally consistent with current clinical evidence and support the clinical plausibility of the model-based simulations.

The models also indicated differences according to prior treatment status within the TPS ≥ 50% subgroup. The median OS was higher in treatment-naïve than in previously treated patients with non-squamous (24.7 vs. 17.1 months) and squamous (20.3 vs. 14.4 months) histology. Although current NCCN guidelines do not explicitly differentiate treatment recommendations according to prior therapy status, the pooled model suggests that the prior treatment histology may contribute to variation in long-term outcomes [43]. This finding illustrates the potential value of integrative modeling for generating clinically relevant hypotheses that may not be readily evaluated within individual trials.

Building on these findings, we extended the OS analysis beyond 5 years, a time horizon rarely addressed in NSCLC trials. Among treatment-naïve patients with non-squamous histology and TPS ≥ 50%, the projected 7- and 10-year survival probabilities were 23% (95% PI, 19–28) and 18% (95% PI, 14–21), respectively, and were slightly higher than those projected for squamous histology. These estimates should be interpreted as extrapolations beyond the available clinical follow-up rather than directly observed outcomes. Nevertheless, they illustrate the potential value of model-based approaches for characterizing long-term survival with ICIs such as pembrolizumab. Unlike conventional chemotherapy, ICIs may produce durable benefit in a subset of patients, as demonstrated in the long-term follow-up of KEYNOTE-001 and KEYNOTE-024, in which 5-year survival exceeded historical expectations for advanced NSCLC [17,20]. Because randomized trials rarely capture outcomes substantially beyond this time frame, model-based projections may provide additional insight into the potential range of long-term survival.

Evidence from other tumor types further supports the biological plausibility of durable benefit following pembrolizumab treatment. In the 10-year follow-up of the KEYNOTE-006, pembrolizumab maintained a long-term survival benefit in patients with advanced melanoma [44]. Analyses by Noringriis et al. and Van Not et al. similarly reported favorable long-term outcomes following pembrolizumab treatment in melanoma [45,46]. Although findings from melanoma cannot be directly extrapolated to NSCLC, they support the broader observation that PD-1 inhibition may produce prolonged survival in a subset of patients.

Nonetheless, this study had some limitations. First, covariate information was restricted to the level of detail reported in the original publications. Covariate information was limited to reported study-level details; for example, treatment regimens could only be classified as monotherapy or combination therapy without consistently identifying the specific chemotherapy backbone. In addition, because the reconstructed-IPD approach relied on published Kaplan–Meier curves, selective or incomplete reporting of survival figures may have introduced reporting-related bias. Patient-level information on subsequent anticancer therapy and treatment crossover was also not consistently available and therefore could not be incorporated into the models. Consequently, long-term OS estimates may reflect the combined influence of the initial pembrolizumab-based treatment and post-progression treatment exposure. Second, the reconstruction of IPD from published KM curves, although validated, inevitably introduces approximation error and cannot fully capture within-trial heterogeneity. Third, the pooled analysis incorporated trials that differed in study design, population characteristics, treatment setting, and follow-up duration, which may have contributed to residual between-study heterogeneity. Because subgroup-specific survival curves were not consistently reported across trials, conventional sensitivity analyses based on systematic trial exclusion were difficult to apply without substantially altering the composition of the pooled population. As an alternative assessment of cross-trial variability, we evaluated clinically comparable cohorts with directly reported survival outcomes and found no statistically significant evidence of heterogeneity in the examined PD-L1-defined monotherapy subgroups. Finally, parametric TTE modeling requires distributional assumptions, and extrapolations beyond the observed follow-up period are inherently uncertain. Although the selected log-normal model provided the best fit among the candidate distributions, it does not explicitly model a cure or long-term survival plateau. The log-normal distribution implies a monotonically decreasing hazard after an early peak and therefore yields a progressively flattening survival tail, which is qualitatively consistent with the durable benefit observed in a subset of patients, but it does not converge to a non-zero asymptote. Therefore, the 7- and 10-year estimates should be interpreted cautiously as model-based projections, particularly because external validation was limited to shorter-term landmark survival and did not directly assess the extrapolated horizon.

Overall, the integration of reconstructed patient-level data with parametric TTE modeling enabled subgroup-specific characterization of survival outcomes beyond the follow-up available from individual trials. The analysis complements existing evidence on pembrolizumab-treated NSCLC by providing additional insight into long-term survival heterogeneity across clinically relevant populations, thereby supporting a more refined interpretation of treatment outcomes within a precision oncology framework. From a pharmacometric and model-informed drug development perspective, this framework highlights the value of model-based integration of published trial data for quantifying covariate effects and supporting long-term evidence generation. This analytical approach may also be applicable to other therapeutic settings in which long-term patient-level survival data are not publicly available.

## Figures and Tables

**Table 1:** Patient characteristics for overall survival and progression-free survival cohorts.

	OS Cohort (*n* = 7165)	PFS Cohort (*n* = 6730)
Age	64.19 (12.27)	64.30 (12.13)
Race		
Non-Asian	5183 (72.34)	4774 (70.94)
Asian	1983 (27.66)	1956 (29.06)
Sex		
Male	4785 (66.78)	4579 (68.04)
Female	2380 (33.22)	2151 (31.96)
Performance status		
0–1	7093 (99)	6658 (98.93)
2+	72 (1)	72 (1.07)
Smoking status		
Ever	5964 (83.24)	5643 (83.85)
Never	1201 (16.76)	1087 (16.15)
Tumor type		
Non-squamous	4977 (69.46)	4623 (68.69)
Squamous	2188 (30.54)	2107 (31.31)
Metastasis	6837 (95.42)	6397 (95.05)
Brain metastasis	809 (11.29)	811 (12.05)
TPS		
<1%	1525 (21.28)	1453 (21.59)
1–49%	2453 (34.24)	2242 (33.31)
≥50%	3187 (44.48)	3035 (45.10)
Treatment type		
Monotherapy	2687 (37.50)	2107 (31.31)
Combination	4479 (62.50)	4623 (68.69)
Previously treated	2097 (28.85)	1774 (26.36)
EGFR mutation	417 (5.82)	361 (5.36)

Data are median (SD) or *n* (%). OS, overall survival; PFS, progression-free survival; TPS, tumor proportion score.

**Table 2:** Parameter estimates and bootstrap confidence intervals for final overall survival and progression-free survival models.

Parameter	TPS ≥ 50%		TPS 1–49%	
	Final Model Estimate (RSE or CV %)	Bootstrap ^a^ 95% CI (2.5–97.5%)	Final Model Estimate (RSE or CV %)	Bootstrap ^a^ 95% CI (2.5–97.5%)
OS model				
Fixed effects ^b^				
*μ_pop_*	2.58 (2.59)	2.45–2.72	2.48 (1.77)	2.40–2.57
*σ_pop_*	1.16 (3.23)	1.09–1.24	1.24 (2.38)	1.18–1.30
*β_Naïve on μ_*	0.12 (18.9)	0.08–0.17	0.08 (32.5)	0.03–0.13
*β_NSQ on μ_*	0.07 (33.5)	0.02–0.11	-	-
*β_COMB on μ_*	-	-	0.13 (19.0)	0.08–0.18
Random effects ^c^				
*ω^2^_on μ_*	0.31 (32.1)	0.28–0.35	0.18 (17.9)	0.15–0.21
*ω^2^_on σ_*	0.47 (49.6)	0.42–0.53	0.29 (29.6)	0.25–0.33
PFS model				
Fixed effects ^b^				
*μ_pop_*	1.51 (4.38)	1.38–1.64	1.42 (3.43)	1.33–1.52
*σ_pop_*	0.90 (2.29)	0.86–0.94	0.93 (1.79)	0.90–0.96
*β_Naïve on μ_*	0.18 (21.3)	0.11–0.26	0.32 (12.1)	0.25–0.40
*β_NSQ on μ_*	0.10 (34.8)	0.03–0.17	-	-
Random effects ^c^				
*ω^2^_on μ_*	0.66 (74.2)	0.62–0.71	0.57 (62.1)	0.52–0.62
*ω^2^_on σ_*	0.15 (15.0)	0.12–0.19	0.11 (10.6)	0.09–0.13

CV, coefficient of variation; OS, overall survival; PFS, progression-free survival; RSE, relative standard error; TPS, tumor proportion score; CI, confidence interval; *μ_pop_*, population-level mean of log survival time; *σ_pop_*, standard deviation on the log scale; *β_Naïve on μ_*, covariate effect of being treatment-naïve on the *μ*; *β_NSQ on μ_*, covariate effect of having non-squamous histology on the *μ*; *β_COMB on μ_*, covariate effect of receiving combination therapy on the μ. ^a^ 95% confidence interval was obtained from a nonparametric bootstrap with 5000 resampling times. ^b^ Fixed effects and residual variability are presented as estimates with RSE %. ^c^ Random effects are presented as estimates with CV %.

**Table 3:** External Validation of the Simulated OS Model Using the LEAP-006 and LEAP-008 Clinical Trials.

Time Point (Months)	LEAP-006		LEAP-008	
	Observed OS (%)	Model-Derived OS (%)	Observed OS (%)	Model-Derived OS (%)
12	69.5	67.6	46.5	51.3
24	45.2	46.6	21.1	23.6
36	36.5	35.0	16.8	17.3

OS, overall survival.

**Table 4:** Long-term overall survival probabilities simulated from the final time-to-event models in treatment-naïve patients in the tumor proportion score ≥ 50% or 1–49% subgroup.

OS Probability (%, 95% PIs)	TPS ≥ 50%		TPS 1–49%	
	Squamous	Non-Squamous	Monotherapy	Combination
5-year OS	25 (21–29)	29 (24–33)	15 (12–19)	23 (18–27)
7-year OS	19 (15–23)	23 (19–28)	10 (7.6–13)	16 (14–20)
10-year OS	15 (11–18)	18 (14–21)	7.2 (4.9–8.7)	11 (10–14)

OS, overall survival; TPS, tumor proportion score; PI, prediction interval.

## Data Availability

The datasets used and/or analyzed during the current study are available from the corresponding author on reasonable request.

## Supplementary Materials

The following supporting information can be downloaded at: https://www.mdpi.com/article/10.3390/pharmaceutics18091177/s1, Figure S1: PRISMA flow diagram of study selection for the systematic review of trials of pembrolizumab in non-small cell lung cancer; Figure S2: Risk of bias assessment of included randomized trials using the Cochrane RoB 2.0 tool; Figure S3: Visual predictive check for the final overall survival and progression-free survival models in tumor proportion score subgroups; Figure S4: Simulated overall survival curves stratified by histology and treatment regimen for the tumor proportion score <1% models; Figure S5: Simulated progression-free survival curves stratified by histology and treatment regimen based on covariate-specific final models; Figure S6: Simulated PFS curves based on the covariate-specific final models for the tumor proportion score <1% subgroup; Table S1: PICOS-SD framework for systematic literature search; Table S2: Parametric survival functions evaluated in the time-to-event analysis; Table S3: Summary of included clinical trials for time-to-event modeling; Table S4: Methodological quality assessment of included studies using the Methodological Index for Non-Randomized Studies (MINORS) tool; Table S5: Parameter estimates and bootstrap confidence intervals for the final overall survival and progression-free survival models in the tumor proportion score <1% subgroup; Table S6: Patient characteristics for the external validation cohorts (LEAP-006 and LEAP-008); Table S7: Long-term overall survival probabilities simulated from the final time-to-event models in previously treated patients in the tumor proportion score ≥50% or 1–49% subgroup. Refs. [47,48,49,50,51,52,53,54,55,56,57,58,59,60,61,62,63,64,65,66,67,68,69,70,71,72,73,74,75,76,77] are cited in the Supplementary Materials.
