# Supplementary material for: Long-Term Survival Heterogeneity Across PD-L1-Defined Subgroups in Pembrolizumab-Treated Non-Small-Cell Lung Cancer: A Model-Based Analysis
Source: Pharmaceutics. 2026 Sep 17;18(9):1177. doi: 10.3390/pharmaceutics18091177 (PMC13610843; doi:10.3390/pharmaceutics18091177)
Supplement: Supplementary file 1 [file pharmaceutics-18-01177-s001.zip › pharmaceutics-4511525-supplementary.pdf]

Supplementary information

Table S1. PICOS-SD framework for systematic literature search

Table S2. Parametric survival functions evaluated in the time-to-event analysis

Table S3. Summary of included clinical trials for time-to-event modeling

Table S4. Methodological quality assessment of included studies using the Methodological Index for Single-Arm Studies (MINORS) tool

Table S5. Parameter estimates and bootstrap confidence intervals for the final overall survival and progression-free survival models in the tumor proportion score <1% subgroup

Table S6. Patient characteristics for the external validation cohorts (LEAP-006 and LEAP-008)

Table S7. Long-term overall survival probabilities simulated from the final time-to-event models in previously treated patients in the tumor proportion score ≥50% or 1–49% subgroup

Figure S1. PRISMA flow diagram of study selection for the systematic review of trials of pembrolizumab in non-small cell lung cancer

Figure S2. Risk of bias assessment of included randomized trials using the Cochrane RoB 2.0 tool

Figure S3. Visual predictive check for the final overall survival and progression-free survival models in tumor proportion score subgroups

Figure S4. Simulated overall survival curves stratified by histology and treatment regimen for the tumor proportion score <1% models

Figure S5. Simulated progression-free survival curves stratified by histology and treatment regimen based on covariate-specific final models

Figure S6. Simulated PFS curves based on the covariate-specific final models for the tumor proportion score <1% subgroup

Table S1. PICOS-SD framework for systematic literature search

Categories	Contents
P (population)	Adult patients with advanced or metastatic non-small cell lung cancer
I (intervention)	Pembrolizumab (Keytruda®, MK-3475, SCH-900475)<br>Pembrolizumab monotherapy or pembrolizumab-based combination therapy
C (comparator)	Any comparator or no comparator
O (outcome)	Overall survival and/or progression-free survival reported using extractable Kaplan–Meier curves
T (time frame)	No restriction on treatment line or follow-up duration
S (setting)	Advanced NSCLC across treatment lines
SD (study design)	Phase I–III prospective clinical trials, including randomized and single-arm studies

Table S2. Parametric survival functions evaluated in the time-to-event analysis

Distribution	Equation
Weibull distribution	$S(t) = exp(-\lambda t^{\gamma})$
Gompertz distribution	$S(t) = exp\left(-\frac{\lambda}{\gamma}(e^{\gamma t} - 1)\right)$
Generalized gamma distribution	$S(t) = 1 - F\left(\frac{\ln t - \mu}{\sigma}; Q\right)$
Log-logistic distribution	$S(t) = \frac{1}{1 + (\lambda t)^{\gamma}}$
Log-normal distribution	$S(t) = 1 - \phi\left(\frac{\ln t - \mu}{\sigma}\right)$

Where, λ,γ,μ, and σ denote scale, shape, location, and dispersion parameters, respectively. $F(\cdot\,;Q)$ represents the cumulative distribution function (CDF) of the generalized gamma distribution with shape parameter Q and $\phi(\cdot)$ is the standard normal CDF.

Table S3. Summary of included clinical trials for time-to-event modeling

Study (year)	Number and location of centers	Follow-up duration (months)	Study arms	Number of patients	Age and sex (median, %)	Non-squamous cell (%)	TPS ≥50% (%)	Previous therapy (%)
Langer CJ (2016) [47]	26, USA and Taiwan	25	**Arm A** Pembrolizumab 4 cycles, 200 mg + chemotherapy (pemetrexed, carboplatin)	60	Age: 62.5 Male: 37%	97	33	0 (Naïve)
Langer CJ (2016) [47]	26, USA and Taiwan	25	**Arm B** Chemotherapy	63	Age: 63.2 Male: 41%	87	27	0 (Naïve)
Gadgeel SM (2018) [48]	12, USA and Taiwan	35	**Arm A** Pembrolizumab + carboplatin + paclitaxel	25	Age: 66 Male: 48%	52	36	0 (Naïve)
Gadgeel SM (2018) [48]	12, USA and Taiwan	35	**Arm B** Pembrolizumab + carboplatin + paclitaxel + bevacizumab	25	Age: 62 Male: 52%	84	32	0 (Naïve)
Gadgeel SM (2018) [48]	12, USA and Taiwan	35	**Arm C** Pembrolizumab + pemetrexed + carboplatin	24	Age: 59.5 Male: 50%	79	33	0 (Naïve)
Mok TSK (2019) [16]	213, 32 countries	42	**Arm A** Pembrolizumab 200 mg Q3W, 35 cycles	637	Age: 63 Male: 71%	62	47	0 (Naïve)
Mok TSK (2019) [16]	213, 32 countries	42	**Arm B** Carboplatin + paclitaxel or pemetrexed	637	Age: 63 Male: 71%	61	47	0 (Naïve)
Garon EB (2019) [20]	NA	65	**Arm A** Treatment naïve (Pembrolizumab 2 mg/kg or 10 mg/kg Q2W, Q3W)	101	Age: 64 Male: 53%	81	30	71.6
Garon EB (2019) [20]	NA	65	**Arm B** Previously treated (Pembrolizumab 2 mg/kg or 10 mg/kg Q2W, Q3W)	449	Age: 64 Male: 53%	81	30	71.6
Gubens MA (2019) [49]	11, USA	30	**Arm A** Pembrolizumab 10 mg/kg + ipilimumab	6	Age: 64 Male: 50%	67	67	100

			**Arm B**<br>Pembrolizumab 2 mg/kg + ipilimumab	45	Age: 61<br>Male: 51%	82	13	
Nishio M (2019) [50]	NA	36	Pembrolizumab 10 mg/kg Q3W	38	Age: 66<br>Male: 68%	82	32	0 (Naïve)
Levy BP (2019) [51]	33,<br>6 countries	17	Pembrolizumab 200 mg Q3W	100	Age: 66<br>Male: 63.3%	83.7	18.4	100
Theelen WSME (2019) [52]	3,<br>Netherlands	18	Pembrolizumab 200 mg Q3W	76	Age: 62<br>Male: 57%	88	20	100
Gadgeel S (2020) [53]	NA	30	**Arm A**<br>Pembrolizumab combination 200 mg q3w, 35 cycles<br>(combination: pemetrexed and platinum)	410	Age: 65<br>Male: 62%	100	32	0 (Naïve)
			**Arm B**<br>Placebo combination<br>(combination: pemetrexed and platinum)	206	Age: 63.5<br>Male: 53%	100	34	
Goldberg SB (2020) [54]	1,<br>USA	24	Pembrolizumab 10 mg/kg Q2W	42	Age: 60<br>Male: 33%	86	88<br>(TPS≥1%)	0 (Naïve)
Herbst RS (2020) [55]	202,<br>24 countries	55	**Arm A**<br>Pembrolizumab 2 mg/kg or 10 mg/kg Q3W	690	Age: 57.2<br>Male: 62%	70	42	100

			**Arm B**<br>Docetaxel	343	Age: 60.9<br>Male: 61%	70	44	
Arrieta O (2020) [56]	1,<br>Mexico	30	**Arm A**<br>Pembrolizumab 200 mg Q3W + docetaxel	40	Age: 50<br>Male: 48%	93	21	100
			**Arm B**<br>Docetaxel	38	Age: 62<br>Male: 34%	87	38	
Middleton G (2020) [57]	10,<br>UK	25	Pembrolizumab 200 mg Q3W	60	Age: 72<br>Male: 55%	68	25	0 (Naïve)
Jabbour SK (2020) [58]	10,<br>UK	30	Pembrolizumab 200 mg Q3W<br>+ carboplatin + paclitaxel	21	Age: 69.5<br>Male: 48%	52	26	0 (Naïve)
Durm GA (2020) [59]	NA	42	Pembrolizumab 200 mg Q3W	92	Age: 66<br>Male: 64%	55	58.5	100
Reck M (2021) [60]	NA	72	**Arm A**<br>Pembrolizumab 200 mg Q3W, 35 cycles	154	Age: 64.5<br>Male: 59.7%	81.2	100	0 (Naïve)
			**Arm B**<br>Carboplatin + paclitaxel or pemetrexed	151	Age: 66<br>Male: 62.9%	82.1	100	
Horinouchi H (2021) [61]	NA	36	**Arm A**<br>Pembrolizumab combination 200 mg Q3W, 35 cycles<br>(combination: pemetrexed and platinum)	25	Age: 64<br>Male: 76%	92	40<br>(TPS≥1%)	0 (Naïve)

			**Arm B**<br>Placebo combination<br>(combination: pemetrexed and platinum)	15	Age: 66<br>Male: 80%	93	40<br>(TPS≥1%)	
Wu YL (2021) [62]	NA	42	**Arm A**<br>Pembrolizumab combination 200 mg Q3W, 35 cycles<br>(combination: pemetrexed/paclitaxel and platinum)	128	Age: 82<br>Male: 44.5%	56.3	100	0 (Naïve)
			**Arm B**<br>Placebo combination<br>(combination: pemetrexed/paclitaxel and platinum)	134	Age: 88.8<br>Male: 43.3%	55.2	100	
Boyer M (2021) [40]	171,<br>24 countries	33	**Arm A**<br>Pembrolizumab 200 mg Q3W, 35 cycles + ipilimumab	284	Age: 64<br>Male: 64%	72.9	100	0 (Naïve)
			**Arm B**<br>Pembrolizumab + placebo	284	Age: 65<br>Male: 65%	71.5	100	
Masuda T (2022) [63]	NA	36	Pembrolizumab 200 mg Q3W	26	Age: 78<br>Male: 69.3%	69.2	100	0 (Naïve)
Jung HA (2022) [64]	1,<br>South Korea	33	**Arm A**<br>Pembrolizumab combination 200 mg Q3W, 35 cycles<br>(combination: gemcitabine, pemetrexed, docetaxel, vinorelbine)	47	Age: 63<br>Male: 78.7%	57.5	51.1	100
			**Arm B**<br>Placebo combination<br>(combination: gemcitabine, pemetrexed, docetaxel, vinorelbine)	51	Age: 64<br>Male: 84.3%	51	54.9	
Reckamp KL (2022) [65]	1,<br>USA	30	**Arm A**<br>Docetaxel or gemcitabine or pemetrexed	47	Age: 67<br>Male: 63%	58	25	100

			**Arm B**<br>Pembrolizumab + ramucirumab	51	Age: 69<br>Male: 59%	59	19	
Lim SM (2023) [66]	54,<br>12 countries	31	**Arm A**<br>Dostarlimab combination<br>(combination: pemetrexed and platinum)	47	Age: 67<br>Male: 63%	58	25	0 (Naïve)
			**Arm B**<br>Pembrolizumab combination 200 mg Q3W, 35 cycles<br>(combination: pemetrexed and platinum)	51	Age: 69<br>Male: 59%	59	19	
Ren S (2023) [67]	NA	33	**Arm A**<br>Pembrolizumab 2 mg/kg Q3W, 35 cycles	114	Age: 61<br>Male: 79.8%	57.9	100	100
			**Arm B**<br>Docetaxel	113	Age: 63<br>Male: 80.5%	53.1	99.1	
Novello S (2023) [13]	NA	72	**Arm A**<br>Pembrolizumab combination 200 mg Q3W, 35 cycles<br>(combination: carboplatin or paclitaxel)	278	Age: 65<br>Male: 79.1%	2.6	26.3	0 (Naïve)
			**Arm B**<br>Placebo combination<br>(combination: carboplatin or paclitaxel)	281	Age: 65<br>Male: 83.6%	2.5	26	
Yang JC (2023) [41]	158,<br>USA	51	**Arm A**<br>Pembrolizumab combination 200 mg Q3W, 35 cycles<br>(combination: pemetrexed or platinum)	245	Age: 62<br>Male: 38%	100	21.2	100
			**Arm B**<br>Placebo combination<br>(combination: pemetrexed or platinum)	247	Age: 64<br>Male: 38.9%	100	20.6	
Spicer JD (2024) [68]	189,<br>25 countries	66	**Arm A**<br>Pembrolizumab combination 200 mg Q3W, 35 cycles<br>(combination: pemetrexed or platinum)	397	Age: 63<br>Male: 70%	57	33	0 (Naïve)
			**Arm B**<br>Placebo combination<br>(combination: pemetrexed or platinum)	400	Age: 64<br>Male: 71%	57	34	

Shiraishi Y (2024) [69]	48,<br>Japan	30	**Arm A**<br>Pembrolizumab 200 mg Q3W, 35 cycles	147	Age: 68<br>Male: 79%	78	17	0 (Naïve)
			**Arm B**<br>Nivolumab + ipilimumab	148	Age: 68<br>Male: 81%	78	16	
Yang JC (2024) [70]	162,<br>19 countries	24	**Arm A**<br>Pembrolizumab 200 mg Q3W, 35 cycles + Lenvatinib	309	Age: 66<br>Male: 74.4%	62.8	44.3	0 (Naïve)
			**Arm B**<br>Pembrolizumab	314	Age: 66<br>Male: 71.3%	65.6	44.3	
Gentzler RD (2024) [71]	18,<br>USA	48	Pembrolizumab 200 mg Q3W, 34 cycles + paclitaxel	46	Age: 65.5<br>Male: 52%	48	23	0 (Naïve)
Tokito T (2024) [72]	101,<br>18 countries	12	**Arm A**<br>Pembrolizumab 200 mg Q3W, 35 cycles + epacadostat	77	Age: 64<br>Male: 68.8%	62.8	74	0 (Naïve)
			**Arm B**<br>Placebo	77	Age: 69<br>Male: 76.6%	65.6	71.4	
Shin J (2024) [73]	NA	72	Pembrolizumab 200 mg Q3W, ~2 years	37	Age: 63<br>Male: 62.2%	73	2.7	0 (Naïve)
Furqan M (2024) [74]	5,<br>USA	24	Pembrolizumab 2 mg/kg Q3W	30	Age: 66<br>Male: 53.3%	83.3	18.5	0 (Naïve)

Tan DSW (2024) [75]	NA	24	**Arm A**<br>Canakinumab combination<br>(combination: pemetrexed/paclitaxel + platinum)	320	Age: 63<br>Male: 70.9%	69.4	27.8	0 (Naïve)
			**Arm B**<br>Pembrolizumab combination 200 mg Q3W, 35 cycles<br>(combination: pemetrexed/paclitaxel + platinum)	323	Age: 63<br>Male: 71.8%	70	28.2	
Hochmair M (2025) [76]	178,<br>20 countries	48	**Arm A**<br>Pembrolizumab + Olaparib + chemotherapy<br>(chemotherapy: carboplatin or paclitaxel)	296	Age: 65<br>Male: 81.4%	2	28.7	0 (Naïve)
			**Arm B**<br>Pembrolizumab + chemotherapy<br>(chemotherapy: carboplatin or paclitaxel)	295	Age: 64<br>Male: 79.7%	2	28.7	
Gray JE (2025) [77]	178,<br>20 countries	51	**Arm A**<br>Pembrolizumab + Olaparib	337	Age: 63<br>Male: 67.4%	100	32.3	0 (Naïve)
			**Arm B**<br>Pembrolizumab + pemetrexed	335	Age: 62<br>Male: 67.5%	100	32.8	

TPS, tumor proportion score.

Table S4. **Methodological quality assessment of included studies using the Methodological Index for Non-Randomized Studies (MINORS) tool**

Study (year)	Clearly stated aim	Inclusion of consecutive patients	Prospective data collection	Endpoints appropriate to the aim	Unbiased assessment of endpoint	Follow-up period appropriate	Loss to follow-up <5%	Prospective calculation of study size	Total (0–16)
Nishio M (2019)	2	2	2	2	2	2	2	0	14
Levy BP (2019)	2	2	2	2	2	1	2	0	13
Theelen WSME (2019)	2	2	2	2	2	1	2	0	13
Goldberg SB (2020)	2	2	2	2	2	2	2	0	14
Middleton G (2020)	2	2	2	2	2	2	2	1	15
Jabbour SK (2020)	2	2	2	2	2	2	2	1	15
Durm GA (2020)	2	2	2	2	2	2	0	0	12
Masuda T (2022)	2	2	2	2	2	2	2	1	15
Gentzler RD (2024)	2	2	2	2	2	2	2	0	14
Shin J (2024)	2	2	2	2	2	2	2	2	16
Furqan M (2024)	2	2	2	2	2	2	2	1	15

Table S5. Parameter estimates and bootstrap confidence intervals for the final overall survival and progression-free survival models in the tumor proportion score <1% subgroup

**Parameter**	**OS model**		**PFS model**	
	Final model Estimate (RSE or CV %)	Bootstrap[a] 95% CI (2.5%–97.5%)	Final model Estimate (RSE or CV %)	Bootstrap[a] 95% CI (2.5%–97.5%)
Fixed effects[b]				
μ_{pop}	2.35 (3.62)	2.19–2.52	1.50 (4.85)	1.37–1.66
σ_{pop}	1.04 (5.21)	0.94–1.15	0.72 (4.91)	0.66–0.80
$\beta_{\text{Naïve on }\mu}$	0.21 (13.9)	0.16–0.27	0.28 (19.0)	0.17–0.38
$\beta_{\text{NSQ on }\mu}$	0.07 (38.8)	0.02–0.13	-	-
Random effects[c]				
$\omega^2_{\text{on }\mu}$	0.20 (19.8)	0.14–0.27	0.57 (61.8)	0.52–0.63
$\omega^2_{\text{on }\sigma}$	0.39 (40.6)	0.32–0.48	0.29 (29.7)	0.22–0.39

CV, coefficient of variation; OS, overall survival; PFS, progression-free survival; RSE, relative standard error; TPS, tumor proportion score.

μ_{pop}, population-level mean of log survival time; σ_{pop}, standard deviation on the log scale; $\beta_{\text{Naïve on }\mu}$, covariate effect of being treatment-naïve on the μ; $\beta_{\text{NSQ on }\mu}$, covariate effect of having non-squamous histology on the μ.

[a] 95% confidence interval was obtained from a nonparametric bootstrap with 1,000 resampling times.

[b] Fixed effects and residual variability are presented as estimates with RSE %.

[c] Random effects are presented as estimates with CV %.

Table S6. Patient characteristics for the external validation cohorts (LEAP-006 and LEAP-008)

	**LEAP-006 (n = 373)**	**LEAP-008 (n = 185)**
Median Age (range)	64 (28–87)	65 (41–81)
Race		
Non-Asian	261 (70.0)	NA
Asian	112 (30.0)	NA
Sex		
Male	247 (66.2)	118 (63.8)
Female	126 (33.8)	67 (36.2)
Smoking status		
Ever	302 (81.0)	NA
Never	71 (19.0)	NA
Tumor type		
Non-squamous	373 (100)	135 (73.0)
Squamous	0	50 (27.0)
Metastasis	373 (100)	185 (100)
TPS		
<1%	121 (32.4)	90 (48.6)
1–49%	148 (39.7)	45 (24.3)
≥50%	91 (24.4)	34 (18.4)
Previously treated	0	185 (100)

Data are n (%).

Table S7. Long-term overall survival probabilities simulated from the final time-to-event models in previously treated patients in the tumor proportion score ≥50% or 1–49% subgroup

OS rate (%, 95% PIs)	TPS ≥50%		TPS 1–49%	
	Squamous	Non-squamous	Monotherapy	Combination
5-year OS	18 (14–22)	21 (15–24)	12 (9–16)	18 (14–23)
7-year OS	14 (10–18)	16 (12–21)	8.3 (5.4–12)	10.3 (7.2–13)
10-year OS	10 (7–14)	12 (9–16)	5.6 (3.4–8.1)	8.4 (6.1–10.4)

OS, overall survival; TPS, tumor proportion score.

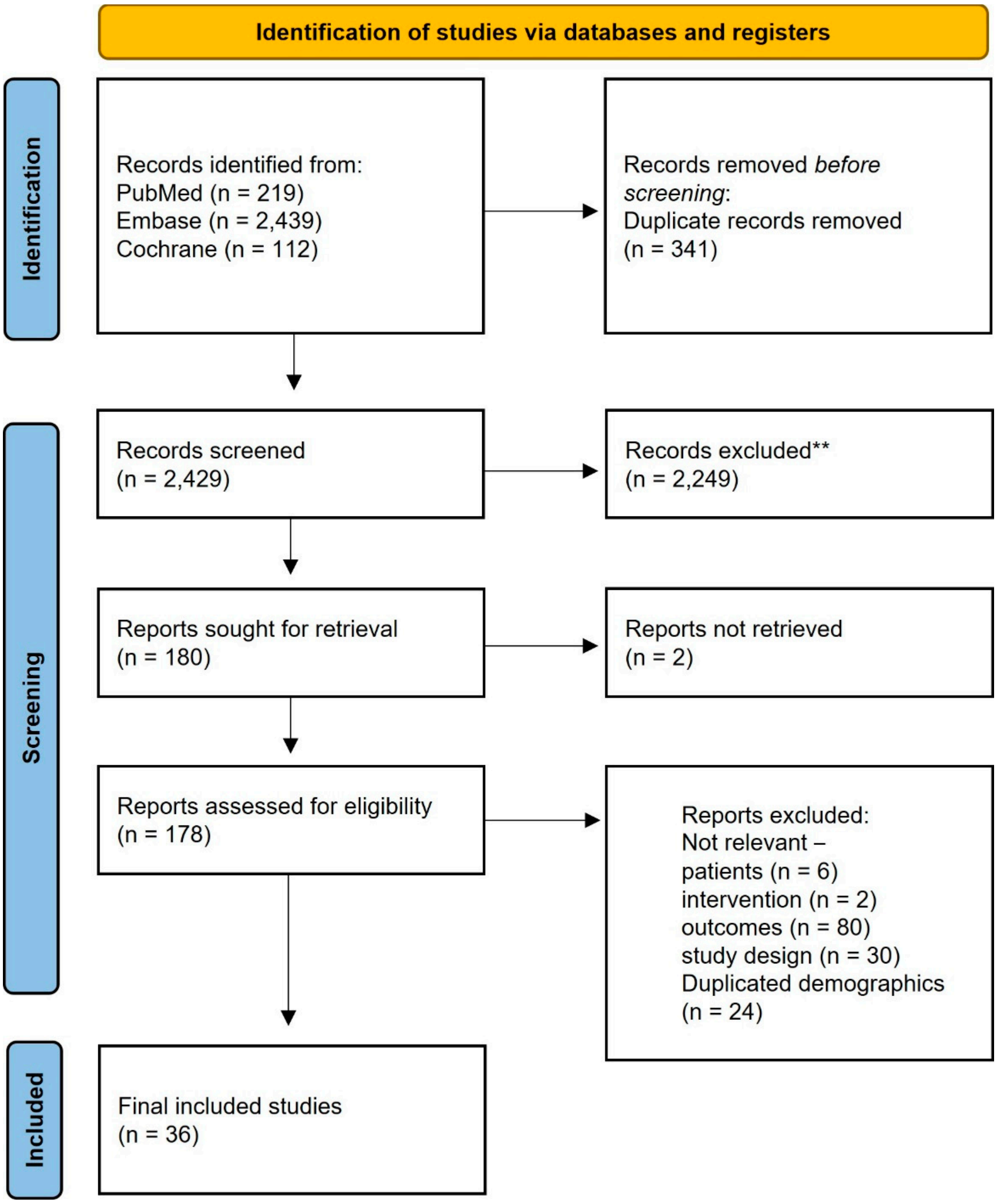

Figure S1. PRISMA flow diagram of study selection for the systematic review of pembrolizumab trials in non-small cell lung cancer.

Unique ID	Study ID	Experimental	Comparator	Weight	D1	D2	D3	D4	D5	Overall
1	Langer CJ (2016)	pembro+chemo	chemo	1	+	+	+	+	+	+
2	Gadgeel SM (2018)	pembro+chemo	chemo	1	!	!	+	!	+	+
3	Mok TSK (2019)	Pembro	chemo	1	+	!	+	+	+	+
4	Garon EB (2019)	previously treated	naive	1	+	!	+	+	+	+
5	Gubens MA (2019)	pembro 10	pembro 2	1	+	+	+	+	+	+
6	Gadgeel S (2020)	pembro	chemo	1	+	+	+	+	+	+
7	Herbst RS (2020)	pembro	docetaxel	1	+	!	+	+	+	+
8	Arrieta O (2020)	pembro+docetaxel	docetaxel	1	+	!	+	+	+	+
9	Reck M (2021)	pembro	chemo	1	+	!	+	+	+	+
10	Horinouchi (2021)	pembro+chemo	chemo	1	+	+	+	+	+	+
11	Wu YL (2021)	pembro combi	chemo	1	+	!	+	+	+	+
12	Boyer M (2021)	pembro+ipi	chemo	1	+	+	+	+	+	+
13	Jung HA (2022)	pembro+chemo	chemo	1	+	+	+	!	+	+
14	Reckamp KL (2022)	pembro+ramucirumab	chemo	1	+	!	+	!	+	+
15	Lim SM (2023)	Pembro combination	Dostarlimab combination	1	+	+	+	+	+	+
16	Ren S (2023)	pembro combi	docetaxel	1	+	!	+	+	+	+
17	Novello S (2023)	Pembro combination	chemo	1	+	!	+	+	+	+
18	Yang JC (2023)	pembro combination	chemo	1	+	+	+	+	+	+
19	Spicer JD (2024)	pembro combination	chemo	1	+	+	+	+	+	+
20	Shiraishi Y (2024)	pembro	nivolumab+ipi	1	+	!	+	!	+	+
21	Yang JC (2024)	lenvatinib+pembro	pembro	1	+	+	+	+	+	+
22	Tokito T (2024)	epacadostat+pembro	chemo	1	+	+	+	+	+	+
23	Tan DSW (2024)	pembro combination	canakinumab combination	1	+	+	+	+	+	+
24	Hochmair M (2025	pembro+olaparib combir	pembro combination	1	+	+	+	+	+	+
25	Gray JE (2025)	pebro+olaparib	pembro combination	1	+	+	+	+	+	+

+ Low risk
! Some concerns
- High risk

D1 Randomisation process
D2 Deviations from the intended interventions
D3 Missing outcome data
D4 Measurement of the outcome
D5 Selection of the reported result

Figure S2. Risk of bias assessment of included randomized trials using the Cochrane RoB 2.0 tool.

Risk of bias was evaluated across five domains using the Cochrane RoB 2.0 tool: D1, randomization process; D2, deviations from the intended interventions; D3, missing outcome data; D4, measurement of the outcome; D5, selection of the reported result. Green circles indicate low risk of bias, yellow circles indicate some concerns, and red circles indicate high risk of bias.

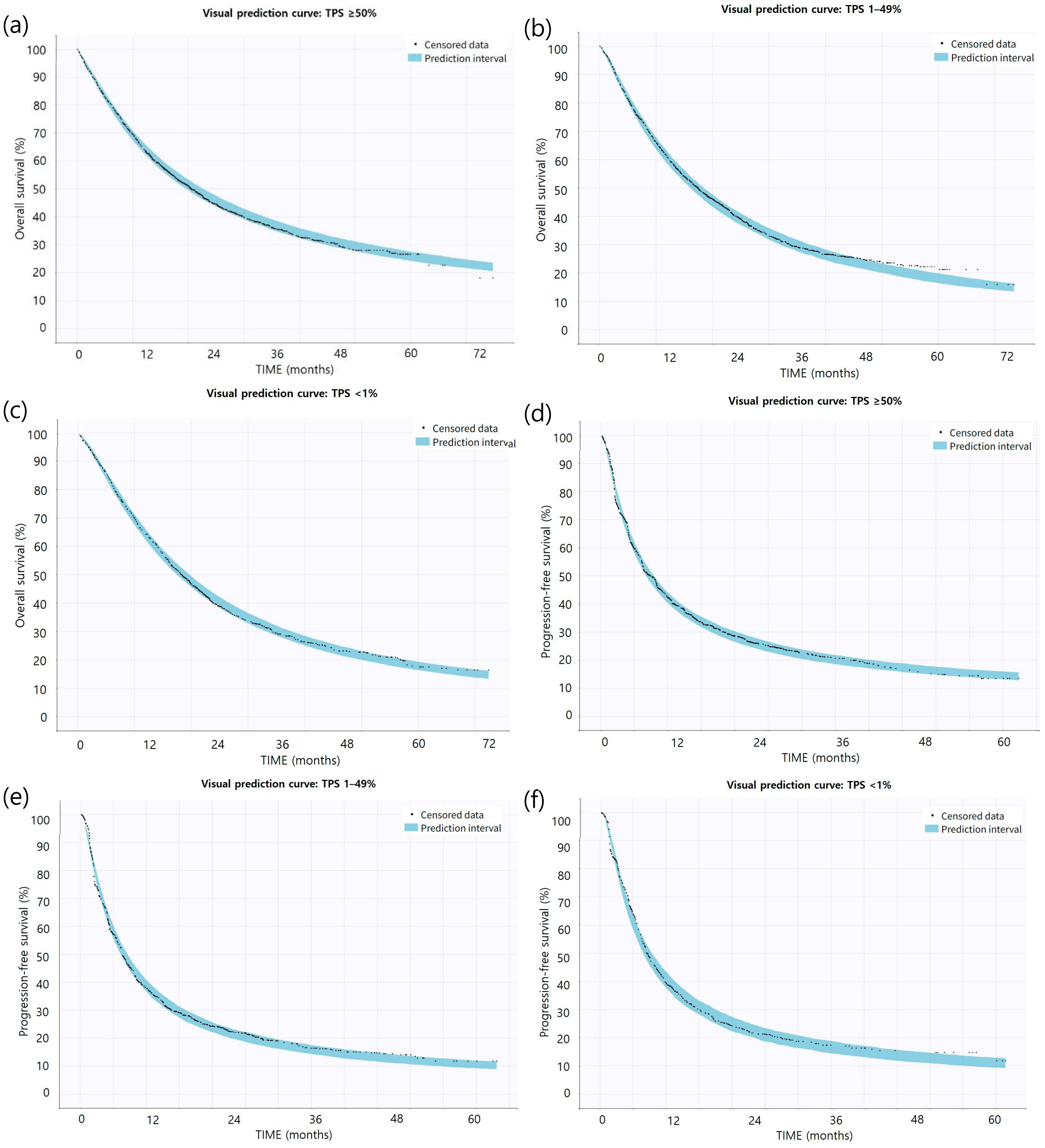

Figure S3. Visual predictive check for the final overall survival and progression-free survival models in tumor proportion score subgroups

Visual predictive checks (VPCs) for the final overall survival (OS) and progression-free survival (PFS) models stratified by PD-L1 TPS subgroups. Panels (a–c) show OS VPCs for TPS ≥50%, TPS 1–49%, and TPS <1%, respectively, while panels (d–f) show the corresponding PFS VPCs. Black dotted lines represent observed survival profiles reconstructed from published clinical trial data, and blue shaded regions represent the 95% prediction intervals derived from repeated model simulations.

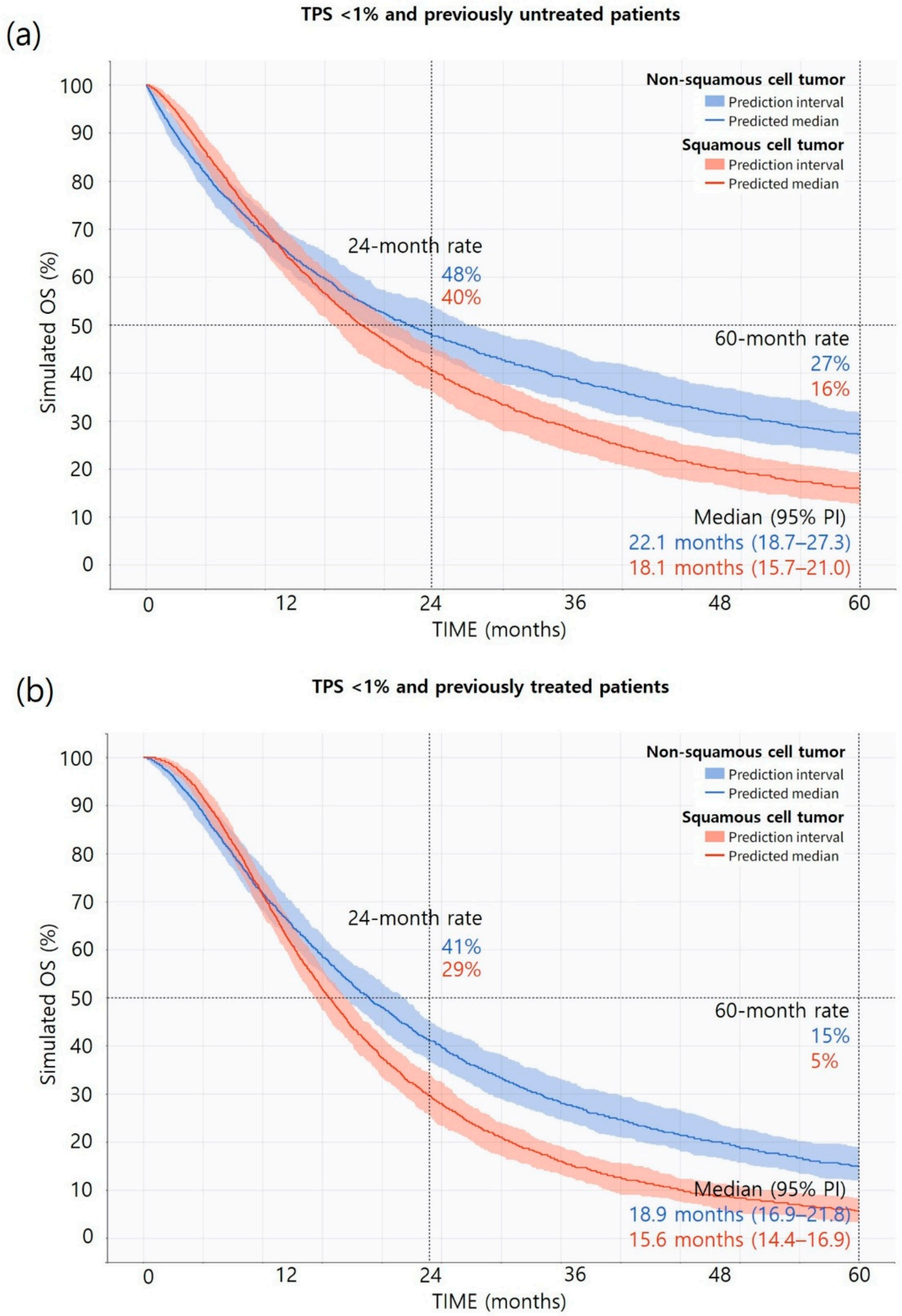

Figure S4. Simulated overall survival curves stratified by histology and treatment regimen for tumor proportion score <1% models.

TPS <1% model stratified by histology for previously untreated (a) and treated (b) patients. Blue and red shaded areas represent 95% prediction intervals for non-squamous vs. squamous. Solid lines indicate median OS. The 24- and 60-month survival rates and median OS (95% PI) are annotated. OS, overall survival; TPS, tumor proportion score.

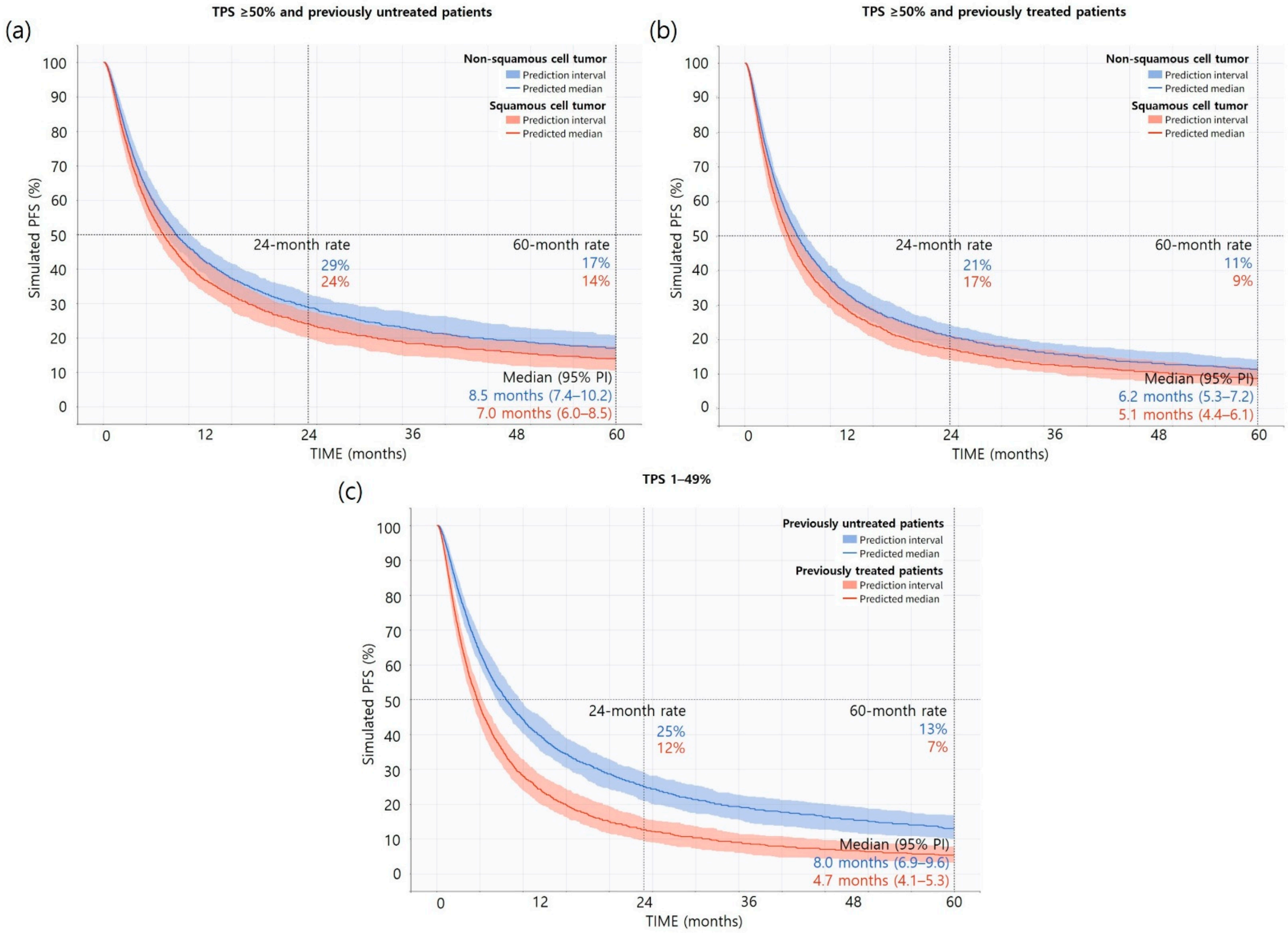

Figure S5. Simulated progression-free survival curves stratified by histology and treatment regimen based on covariate-specific final models.

(a, b) TPS ≥50% model stratified by histology (non-squamous vs. squamous) in treatment-naïve (a) and previously treated (b) patients. (c) TPS 1–49% model stratified by prior treatment status (treatment-naïve vs. previously treated). Blue and red shaded areas represent 95% prediction intervals for non-squamous vs. squamous (a, b) and treatment-naïve vs. previously treated (c), respectively. Solid lines indicate median PFS. Median PFS with 95% PIs and 24- and 60-month survival probabilities are annotated.

PFS, progression-free survival; TPS, tumor proportion score.

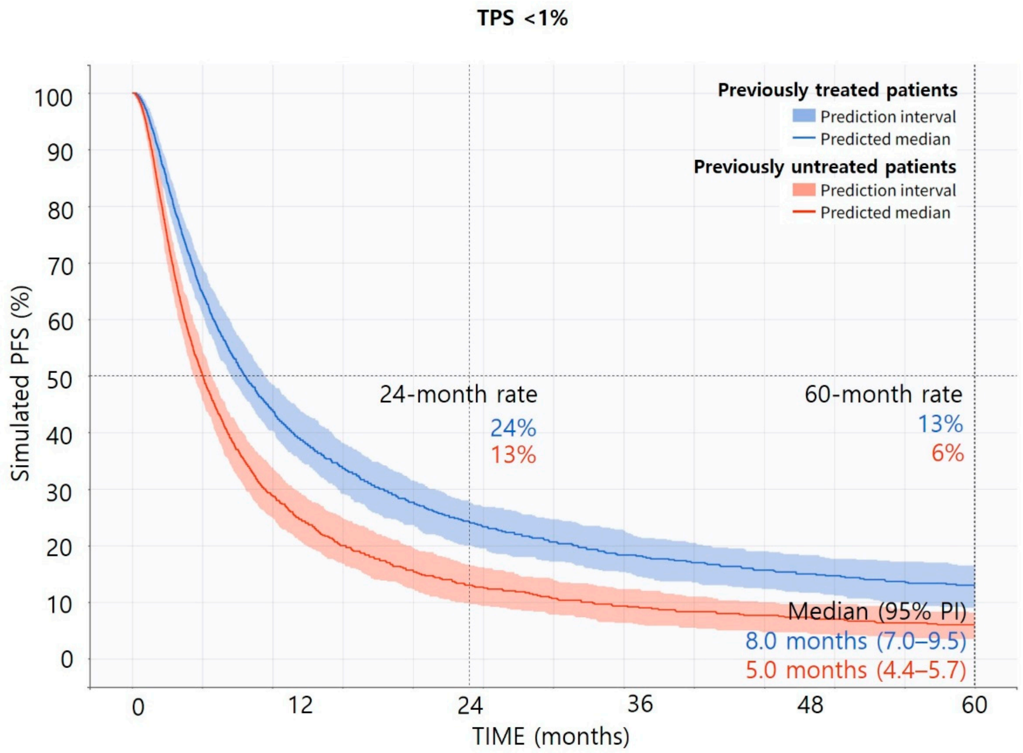

Figure S6. Simulated progression-free survival curves based on the covariate-specific final models for the tumor proportion score <1% subgroup.

Simulated PFS stratified by prior treatment status (treatment-naïve vs. previously treated). Blue and red shaded areas represent 95% prediction intervals. Solid lines indicate median PFS. Median PFS with 95% PIs and 24- and 60-month survival probabilities are annotated.

PFS, progression-free survival; TPS, tumor proportion score.
